# A Retrospective Study of the Career Decision-Making of Young Adults Who Participated in the PSMIGP Program for Gifted Preschoolers

**DOI:** 10.3390/jintelligence14090200

**Published:** 2026-09-01

**Authors:** Chien-Hong Yu, Ching-Chih Kuo

**Affiliations:** 1 Department of Special Education, University of Taipei, Taipei 100234, Taiwan; 2 Department of Special Education, National Taiwan Normal University, Taipei 10610, Taiwan

**Keywords:** gifted education, preschool giftedness, follow-up study, career decision

## Abstract

Gifted students possess broad developmental potential; however, these characteristics may also create challenges in career decision-making. Previous research suggests that gifted individuals’ career development is influenced by multiple interacting factors across personal, familial, educational, and sociocultural domains. This retrospective study investigated the career development of individuals who participated in the preschool gifted program (PSMIGP: Problem-Solving and Multiple Intelligences for Gifted Preschoolers) launched by National Taiwan Normal University in 2003. Semi-structured interviews were conducted with 18 participants selected based on domain of strength, gender, cohort, and willingness to participate. Interview data were analyzed using NVivo 15 through qualitative coding procedures, with multiple data sources, collaborative review, and peer discussion employed to enhance trustworthiness and contextual understanding. Findings revealed that gifted individuals’ career decisions were shaped by interacting personal, family, learning, and sociocultural factors. Although participants across domains demonstrated strong self-awareness and agency, academic participants generally followed more stable career pathways, whereas artistic participants encountered greater complexity and practical constraints. Artistic participants also exhibited greater sensitivity to perceived sociocultural risks. Based on these findings, this study underscores the importance of parental support, strengthened career guidance, and diverse exploratory opportunities within educational settings and the promotion of diverse social values to support gifted students’ career development. Overall, gifted career development should be understood as a dynamic process shaped through continuous interaction between individuals and their broader developmental contexts.

## 1. Introduction

Twenty years ago, in 2003, the Special Education Center at National Taiwan Normal University launched Taiwan’s first preschool gifted program. The participants were selected when they scored higher than the 93rd percentile on PIQ, VIQ, or FIQ on the Chinese version of the Wechsler Preschool and Primary Scale of Intelligence, Revised Edition (WPPSI-R). A series of observations on the performance of the preschoolers participating in the designed courses (observation activities) were involved in the identification. Considering the limitations of the use of standardized tests, especially for young children, the young children’s portfolios provided by special talents were also used to determine eligibility for the gifted and talented program. Our enrichment program for cultivating problem-solving abilities and multiple intelligences for gifted preschoolers was developed based on the DISCOVER (Discovering Intellectual Strengths and Capabilities while Observing Varied Ethnic Responses) model (Kuo et al., 2010). DISCOVER was developed by June Maker and her colleagues at the University of Arizona in 1987. Problem-solving training is highlighted in the DISCOVER program; this training consists of five different problem types. Six themes were woven into the target goal for the classroom activities: relationship, pattern, change, individuality, cycles, and environment (Kuo et al., 2011). Enrichment classes were held on Saturdays, offering problem-solving, group activities, specialized learning, and self-chosen tasks (Kuo et al., 2011). Over four cohorts, 73 children participated; many of these children were later recognized publicly for achievements such as performances or international awards (C.-H. Yu, 2025; C.-H. Yu & Kuo, 2026).

Two decades have since passed, raising an important question: How are these once-promising children doing today? Among gifted individuals who initially demonstrated similarly high levels of ability, what factors have contributed to differences in their long-term career development? These questions have long attracted the attention of gifted education researchers and practitioners.

More than twenty years have passed since the implementation of the program, a period sufficient for participants to progress from early childhood into adulthood and complete most of their formal education. These individuals are now approximately 22 to 27 years old, with some having entered the workforce and others continuing into higher education or advanced professional training. Reflecting on the development of gifted education in Taiwan, Wu (2013) similarly raised the question, “Where are the gifted individuals of the past now?” and documented the developmental stories of several talented individuals, highlighting diverse pathways of gifted development.

However, beyond documenting outcomes alone, understanding gifted individuals’ developmental trajectories requires examining the broader processes through which those outcomes emerge. For children identified as gifted in early childhood, the transition from potential to adult career development is shaped not only by individual ability but also by the continuous interaction of personal, familial, educational, and sociocultural influences across time. Although many gifted individuals demonstrate similar levels of early promise, their adult career pathways often diverge substantially, suggesting that gifted development is shaped by more than ability alone.

Given the complexity of gifted career development and the limited longitudinal research examining how multiple developmental systems shape career outcomes over time, this study adopts a systemic and developmental perspective to investigate the long-term career development of individuals who participated in a preschool gifted program. Specifically, this study addresses the following research questions:

1. What factors across personal, familial, educational, and sociocultural systems influence gifted individuals’ career decision-making?
2. What similarities and differences exist between academically and artistically gifted individuals in their career decision-making processes?
3. How do gifted individuals negotiate personal aspirations and contextual constraints in constructing their career pathways?

## 2. Literature Review

### 2.1. Theoretical Integration of Career Development Perspectives

Career development has long been conceptualized as a dynamic and lifelong developmental process involving the integration of multiple life roles, occupational experiences, and evolving self-concepts across the lifespan (Super, 1976, 1980). Early career development theories largely emphasized gradual maturation and adaptation across developmental stages. For example, Super’s life-span, life-space theory posits that individuals continuously revise their self-concepts throughout different life stages and role experiences and that career choice reflects the implementation of self-concept into vocational roles. The theory further suggests that individuals encounter distinct developmental tasks and adaptive demands during the stages of growth, exploration, establishment, maintenance, and decline; thus, career development should be understood as a continuous developmental process spanning the entire life course rather than the outcome of a single stage (Super, 1980).

Similarly, Ginzberg (1972) proposed that career decision-making is not a single point-in-time choice but rather a progressive developmental process through which individuals gradually reconcile their interests, abilities, values, and realistic constraints. According to this theory, individuals move through fantasy, tentative, and realistic stages before forming increasingly realistic and stable occupational choices, indicating that career decision-making is developmental and continuous in nature.

As theoretical perspectives evolved, scholars gradually moved beyond traditional stage-based viewpoints and instead emphasized the dynamic, adaptive, and constructive nature of career development. Tiedeman and O’Hara (1963) argued that career development is a continuous decision-making process in which individuals repeatedly differentiate and integrate self-concepts through cycles of anticipation and implementation in response to environmental changes. Extending this perspective, Savickas (2005), through career construction theory, further proposed that individuals are not passive responders to environmental demands but actively construct career meaning and vocational direction through life narratives and subjective interpretation of experiences. In this sense, career is not merely the outcome of occupational choice but also an important medium through which individuals construct self-identity and life stories.

In addition, some theories further highlight the critical role of environmental influences in career development. Krumboltz’s social learning theory proposes that career choices are significantly shaped by learning experiences, environmental conditions, and chance events and introduced the concept of planned happenstance to explain how unexpected opportunities and accidental events may become influential factors in career development (Mitchell et al., 1999). Meanwhile, Holland’s person–environment fit theory approaches career development from the perspective of congruence between individuals and environments, suggesting that career choices are strongly influenced by the degree of fit between personality characteristics and occupational environments and that individuals tend to select work environments compatible with their personality in pursuit of greater job satisfaction and adjustment (Nauta, 2010).

Taken together, contemporary career development theories have gradually shifted from emphasizing stage-based maturation and rational decision-making toward viewing career development as a complex process through which individuals continuously interact with their environments, construct meaning, and dynamically adapt throughout the lifespan. Therefore, career decision-making should not be understood as a singular, linear, or purely rational event but rather as a multidimensional developmental process shaped by the interaction of personal characteristics, environmental contexts, opportunity structures, and subjective meaning-making.

### 2.2. Developmental Characteristics and Career Decision-Making Challenges of Gifted Students

Although the aforementioned theories provide important frameworks for understanding the career development of the general population, gifted students often exhibit a greater degree of complexity, nonlinearity, and uncertainty in their career development processes due to their unique ability structures, psychological characteristics, and developmental trajectories. Compared with general students, gifted students typically demonstrate higher cognitive abilities, rapid learning capacity, superior problem-solving skills, abstract reasoning ability, and strong intellectual curiosity (Subotnik et al., 2011). In addition, many gifted students display high levels of autonomy, intrinsic motivation, creativity, and a strong desire for self-actualization (Neihart et al., 2002). However, while these strengths enhance developmental potential, they may also lead gifted students to encounter more complex psychological and decision-making challenges when facing educational and career choices.

Among these challenges, multipotentiality has been regarded as one of the most representative core issues in gifted career development. The concept of multipotentiality was first introduced by Frederickson and Rothney (1972), referring to highly able individuals’ capacity to pursue multiple career pathways. Subsequent research further indicated that such characteristics may contribute to career indecision and decision-making anxiety among gifted students (Kerr & Sodano, 2003). Kerr and Sodano (2003) suggested that multipotentiality may lead gifted students to experience decision delay, choice anxiety, career indecision, and prolonged exploration. Some students even continue postponing career decisions because they struggle to accept that choosing one path necessarily implies relinquishing other possibilities. Emmett and Minor (1993) similarly found that multipotential gifted students often demonstrate psychological resistance toward committing to a single occupation, as they may perceive such commitment as limiting their overall developmental potential.

In addition to multipotentiality, perfectionism is another common and important psychological characteristic among gifted students. Neihart (1999) noted that many gifted students develop significant perfectionistic tendencies and excessive self-expectations due to prolonged exposure to environments characterized by high expectations, high standards, and high achievement demands. Flett and Hewitt (2002) further argued that highly perfectionistic individuals tend to establish unrealistically high standards and experience strong anxiety regarding mistakes and failure, which may further affect decision-making quality and psychological adjustment. In career choice contexts, this characteristic may lead gifted students to pursue the “best” or “perfect” option rather than the “appropriate” or “feasible” one, thereby increasing decision-making pressure and difficulty.

Kelly and Colangelo (1990) found that many gifted students exhibit excessive caution and repeated hesitation in career selection because they fear wasting their potential, failing to maximize their abilities, or not fulfilling family and societal expectations. Some gifted students may even experience “analysis paralysis” due to excessive consideration of all possible outcomes, making it difficult to reach definitive decisions (Kerr & Sodano, 2003).

Furthermore, gifted students’ career development is deeply influenced by identity development processes. Kerr and Erb (1991) suggested that gifted students often confront identity formation questions such as “Who am I?” and “Who do I want to become?” during adolescence and emerging adulthood. When their multiple abilities conflict with external expectations, identity confusion and role conflict may arise. Recent studies have also indicated that gifted students’ career decisions are influenced not only by ability and interests but also by self-identity, perceived social expectations, family values, and considerations regarding future quality of life. Particularly for twice-exceptional students and gifted students with artistic talents or interdisciplinary strengths, the career decision-making process may become even more complex. These students must often balance multiple talents while negotiating among personal passion, practical economic concerns, family expectations, and societal values (Chu et al., 2024; C.-H. Yu, 2025).

Overall, gifted students’ career development should not be simplified as a linear process in which high ability naturally leads to high achievement. Rather, their career decision-making processes should be understood as highly complex developmental phenomena shaped by the interaction among multiple abilities, psychological characteristics, self-identity, and environmental influences.

### 2.3. The Role of Significant Others and Social Context in Gifted Career Development

Previous studies have indicated that gifted individuals’ career development is deeply influenced by significant others and social contexts. Freeman (2013) noted that family, school, and broader social environments jointly constitute important support systems in gifted students’ development, exerting profound influence on talent development and career choice.

First, the family system is regarded as the earliest and most important source of influence in gifted development. Bloom (1985) suggested that parents often play the role of stimulating interests and providing resources and support during the early stages of talent development. Snowden and Christian (1999) found that parents of gifted children are more likely to encourage creativity, provide enriched environments, and actively participate in their children’s learning processes, all of which help gifted students establish higher self-efficacy and developmental motivation.

Second, schools and teachers are also important developmental supports for gifted students. In addition to providing knowledge and skill training, teachers may influence students’ self-concepts and career expectations through expectations, affirmation, and opportunities offered (Olszewski-Kubilius et al., 2015). At advanced stages of talent development, mentors and professional instructors play particularly critical roles in helping students establish professional identities and future directions (Subotnik et al., 2011).

In addition, role models and sociocultural exemplars may shape gifted students’ career beliefs through observational learning. According to (Bandura, 1986) social cognitive theory, individuals construct self-expectations and career images by observing others’ behaviors and outcomes. Studies have also found that role model interventions and exposure to exemplary figures can improve gifted students’ career self-efficacy, career clarity, and future aspirations (Chen, 2014).

Overall, gifted students’ career development is not solely the result of internal ability but, rather, is built upon the interaction of family, educational, and sociocultural support systems.

### 2.4. A Systems Perspective on Gifted Career Development

In recent years, scholars have increasingly challenged traditional reductionist and linear causal models, advocating for ecological and systems-based perspectives in understanding human development. Bronfenbrenner (1979) proposed ecological systems theory, which suggests that individual development occurs within multiple nested systems—including family, school, peers, institutions, and sociocultural environments—and that these systems interact to jointly influence development.

Fischer and Bidell (2006) similarly argued that development should be understood as a dynamic constructive process resulting from continuous interaction between individuals and contexts rather than as linearly determined by isolated factors. This perspective emphasizes complexity, nonlinearity, and contextual dependence in development.

Applying this perspective to gifted education, Dai (2017) proposed evolving complexity theory (ECT), arguing that talent development is an open, adaptive, and increasingly differentiated process. This theory suggests that individuals continuously adjust and reorganize their abilities through interactions with environmental, cultural, domain-specific, and temporal contexts, ultimately forming complex and highly individualized developmental pathways.

Therefore, gifted career development should be understood as a systemic process shaped by the interaction of multiple levels of influence. Developmental trajectories are jointly affected by personal characteristics, family support, educational resources, cultural values, and social systems rather than being determined solely by intelligence or innate giftedness.

Despite previous studies having examined career development and gifted education from multiple perspectives, several limitations remain. First, most studies tend to examine career theories, gifted traits, or environmental support factors separately, lacking integrative frameworks capable of explaining the multifaceted interactive processes involved in gifted students’ career development (Savickas, 2005; Fischer & Bidell, 2006).

Second, prior research has often focused on a single developmental stage or specific gifted populations, with relatively limited attention given to gifted students’ complete career development trajectories from a longitudinal lifespan perspective (Freeman, 2013).

Third, relatively few longitudinal studies have investigated students identified as gifted during preschool and their long-term career development and adult career decision-making processes. In particular, there is limited research exploring how individuals integrate personal characteristics, family influences, educational experiences, and sociocultural contexts over time to form career directions.

Accordingly, the present study adopts evolving complexity theory (ECT) as its primary theoretical framework to investigate gifted individuals’ career development from childhood to adulthood. ECT provides an integrative developmental systems perspective capable of explaining how gifted individuals’ career trajectories emerge through ongoing interactions among personal characteristics, family influences, educational experiences, and sociocultural contexts across time. Unlike traditional theories that conceptualize giftedness as a fixed individual trait or regard career development as a linear progression, ECT emphasizes that talent development is dynamic, adaptive, nonlinear, and context-dependent. From this perspective, career development is not simply the result of innate ability or rational decision-making but, rather, a developmental process shaped through continuous negotiation between individuals and multiple environmental systems.

ECT is particularly suitable for the present study because it highlights how developmental pathways may diverge even among individuals who initially demonstrate similar levels of gifted potential. Through ongoing interactions with opportunities, constraints, cultural expectations, educational environments, and significant others, gifted individuals gradually construct different career identities and developmental trajectories across the lifespan. This perspective closely aligns with the retrospective and longitudinal nature of the present study, which seeks to understand how early gifted potential evolves into adult career development over time and how multiple systemic factors interact to shape long-term developmental outcomes.

Within this overarching framework, the other career development theories reviewed in this study are used as complementary perspectives to explain specific dimensions of career development. For example, Super’s “life-span” perspective helps explain career development as a lifelong developmental process; Savickas’ career construction theory contributes to understanding identity construction and meaning-making; Krumboltz’s social learning perspective helps interpret the influence of learning experiences, opportunities, and unexpected events; and Bronfenbrenner’s ecological systems theory provides an important conceptual foundation for understanding the multilayered environmental systems embedded within ECT. Together, these perspectives support a more comprehensive understanding of gifted individuals’ career development across time, contexts, and developmental systems.

## 3. Materials and Methods

### 3.1. Research Design

The present study adopted a retrospective design to investigate how various people, experiences, and contextual factors shaped gifted individuals’ career development trajectories from childhood to adulthood. Because each participant possessed a unique developmental and career narrative, each case was viewed as a bounded developmental system in which endogenous and exogenous factors interacted within specific contexts to produce both short- and long-term developmental outcomes. Through in-depth examination of multiple cases, this approach sought to understand participants’ unique developmental experiences while preserving the complexity of their individual life histories. At the same time, cross-case comparisons enabled the identification of recurring patterns and broader developmental themes grounded in participants’ lived experiences rather than isolated life fragments (Dai & Li, 2023; Gierczyk & Pfeiffer, 2021).

This study first employed questionnaires to collect participants’ basic background information and their willingness to participate in follow-up interviews. Semi-structured interviews were then conducted to allow participants to describe their developmental experiences and career decision-making processes as fully as possible. Depending on participants’ preferences, interviews were conducted either face-to-face or online. Each interview lasted approximately 60–90 min. Three participants participated in a second interview because of time limitations during the initial interview or because they wished to provide additional reflections and information afterward. In addition, the mothers of two participants also joined the interviews to enhance the completeness and contextual richness of the research data.

To enhance the depth and contextual understanding of the findings, multiple forms of data and informants were incorporated to corroborate and enrich participants’ developmental narratives. These sources included interviews with different informants, reflective records, and materials provided by participants, such as creative works, awards, media reports, and other relevant documents. Multiple data collection procedures were also employed, including questionnaires, semi-structured interviews, reflective narratives, and document analysis. Rather than serving as a strict methodological triangulation, these diverse sources and procedures were intended to provide greater contextual richness and support a more comprehensive interpretation of gifted individuals’ developmental experiences and career pathways within their individual, contextual, and sociocultural environments.

### 3.2. Participants

The participants in this study were individuals who had taken part in the PSMIGP (Problem-Solving and Multiple Intelligences for Gifted Preschoolers) program, a preschool gifted program developed by the Special Education Center at National Taiwan Normal University. The program was designed for preschool children aged 4–6 and aimed to identify and nurture young children who demonstrated outstanding potential or performance across diverse domains of multiple intelligences, including both gifted and twice-exceptional preschoolers.

**Figure 1:**
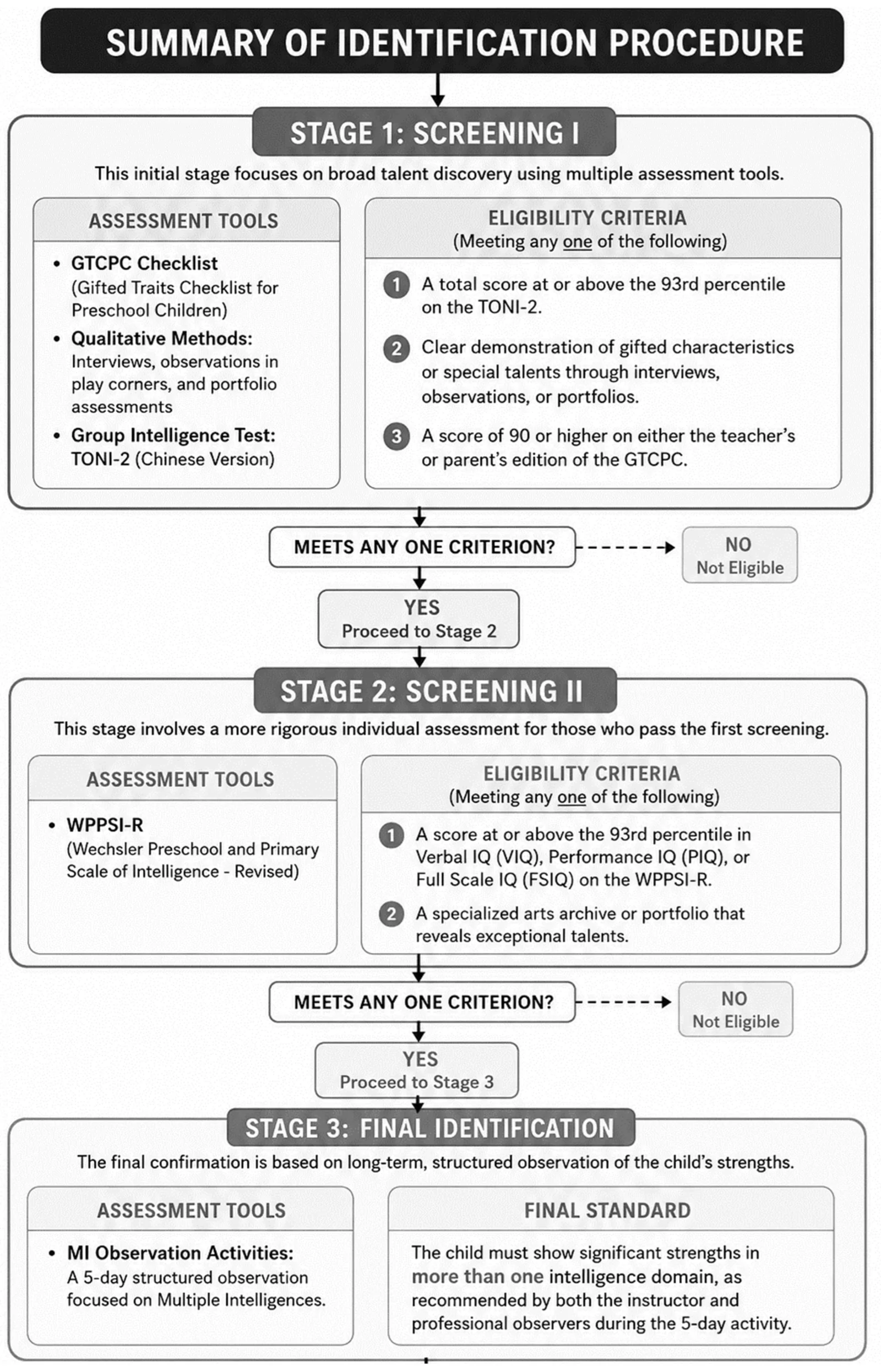
Student identification procedure for the preschool gifted. Adapted from Kuo et al. (2010).

In response to the developmental characteristics of gifted and twice-exceptional preschoolers, the program adopted a multistage and multidimensional identification process to accurately identify children’s strengths and developmental potential. A three-stage identification process was implemented, incorporating multiple assessment methods, including intelligence tests, portfolio assessments, classroom observations, parent interviews, and observational nominations. Rather than relying solely on standardized intelligence test scores, the program emphasized a holistic evaluation of children’s cognitive, creative, artistic, and problem-solving abilities. Participants were subsequently identified and categorized into different domains of strength based on these comprehensive assessments (see Figure 1).

The program was implemented across four cohorts with a total of 73 participating children. Each cohort participated in a 14-week enrichment curriculum conducted on Saturdays throughout the day. The morning sessions primarily focused on exploratory courses integrating multiple intelligences and problem-solving activities, as well as group interaction and collaborative learning activities. The afternoon sessions emphasized domain-specific talent development courses and self-selected enrichment activities, allowing children to further develop their strengths, interests, and autonomous learning experiences. Through this curriculum structure, the program aimed to provide developmentally appropriate opportunities for gifted preschoolers to explore their abilities, enhance creativity and problem-solving skills, and develop their individual talents in diverse domains.

Participants were purposively selected based on their willingness to participate, as well as on considerations of gender, cohort, and domain of strength, in order to ensure diversity in developmental experiences and perspectives across cases. A total of 18 participants were invited for interviews, including nine participants from academic domains and nine from artistic domains. Interviews and data analysis were conducted concurrently, and the interview process continued until recurring patterns and themes became sufficiently established across participants, and no substantially new conceptual categories emerged from subsequent interviews. The background information of the participants is summarized in Table 1.

### 3.3. Instruments

The research instrument used in this study was a semi-structured interview protocol covering the influences of personal, family, learning system, and sociocultural system factors on career development. To ensure the appropriateness of the instrument, five professors from departments of special education were invited to conduct an expert validity review, and revisions were made based on their feedback.

This article represents a partial adaptation of one of the authors’ doctoral dissertation. The original study also explored gifted education experiences and talent development, and those findings have been published in other journals. Therefore, the present study focuses specifically on analyzing the interview questions related to career decision-making. The interview protocol is presented below and is organized into three dimensions: structure, process, and time. These dimensions were designed to explore what factors within the personal, family, learning, and sociocultural systems influence career decision-making, as well as how these factors shape individuals’ career development.

#### 3.3.1. Structural Principle

1. What abilities, characteristics, and opportunities do you personally possess? Please explain which of these have contributed to the development of your talents.
2. Have these abilities, characteristics, and opportunities changed throughout your development? Please describe these changes.
3. How have your abilities, characteristics, and opportunities influenced your future and career decision-making? Please explain how these influences occurred.
4. Has your sociocultural background influenced your talent development and career decision-making?

#### 3.3.2. Process Principle

1. Within learning systems (e.g., schools), how have they influenced your talent development? What important people, events, or experiences have influenced your talent development?
2. Within learning systems (e.g., schools), how have they influenced your career decision-making? What important people, events, or experiences have influenced your career decision-making?
3. Within family systems (e.g., parental educational background, family structure and size, family educational resources), how have they influenced your talent development? What important people, events, or experiences have influenced your talent development?
4. Within family systems (e.g., parental educational background, family structure and size, family educational resources), how have they influenced your career decision-making? What important people, events, or experiences have influenced your career decision-making?
5. Within sociocultural systems (i.e., environments that continuously influence and shape members’ cognitive development, value judgments, behavioral patterns, and psychological processes), how have they influenced your talent development? What important people, events, or experiences have influenced your talent development?
6. Within sociocultural systems (i.e., environments that continuously influence and shape members’ cognitive development, value judgments, behavioral patterns, and psychological processes), how have they influenced your career decision-making? What important people, events, or experiences have influenced your career decision-making?

#### 3.3.3. Temporal Principle

1. When do you think your potential first began to emerge?
2. When do you think your potential developed into ability? Why?
3. When do you think your ability developed into expertise? Why?
4. When do you think your expertise reached excellence? Why?
5. How long has your development in your talent domain continued?
6. When did you decide on your career development direction? Why?
7. Across different developmental stages, what different developmental priorities or important people, events, or experiences do you think influenced you?

### 3.4. Data Analysis

The data in this study were analyzed using thematic analysis with the support of NVivo 15 qualitative analysis software. To ensure systematic organization and interpretation of the qualitative data, coding procedures, including verbatim transcription, open coding, axial coding, and selective coding, were employed to identify, compare, and interpret recurring themes, patterns, and developmental meanings across participants’ developmental experiences and career narratives. In particular, the analysis focused on factors within the personal, family, learning, and sociocultural systems that influenced career development, as well as the interactions between these factors and the individuals themselves.

To enhance the reliability and consistency of the analysis, a doctoral candidate who was also conducting longitudinal follow-up research was invited to serve as a collaborative analyst. Based on the researcher’s coding and analytical interpretations, collaborative discussions and written reviews were conducted to examine coding consistency and interpretive differences. Discrepancies in coding and interpretation were discussed until consensus was reached, after which the findings were further refined and finalized. Reflexive discussions were also conducted throughout the analytical process to reduce potential interpretive bias and strengthen analytical rigor.

### 3.5. Researcher Positionality

One of the authors served as the principal coordinator of the preschool gifted program examined in this study and was extensively involved in its overall planning, curriculum development, and implementation. The other author participated in the program as an instructional teacher, contributing to the design and teaching of enrichment activities within the program. Beyond their involvement in this preschool gifted program, both researchers have long been engaged in gifted education practice, talent development, curriculum design, and follow-up research related to gifted education in Taiwan.

In addition, through their long-term involvement in the preschool gifted program and subsequent educational activities, the researchers maintained ongoing professional relationships with some participants and their parents over many years. These sustained interactions allowed the researchers to develop a deeper contextual understanding of the participants’ developmental backgrounds, educational experiences, family environments, and talent development processes. Such professional familiarity facilitated richer communication during the interviews and enabled more nuanced interpretations of participants’ developmental narratives and career trajectories.

At the same time, the researchers were aware that their prior involvement in the program, longstanding professional experiences in gifted education, and existing relationships with some participants and families could potentially influence data collection and interpretation. Therefore, throughout the research process, reflexive discussions, collaborative coding, peer review procedures, and ongoing analytical reflection were employed to enhance analytical rigor, maintain reflexivity, and reduce potential interpretive bias.

### 3.6. Research Trustworthiness and Ethical Considerations

Interview data were analyzed using NVivo 15 through qualitative coding procedures, with multiple data sources, collaborative review, and peer discussion employed to enhance trustworthiness and contextual understanding. In addition, peer debriefing was conducted by inviting colleagues with backgrounds in gifted education research to review the interview transcripts and coding procedures, clarify similarities and differences in conceptual interpretations, and provide relevant suggestions and revisions. Furthermore, this study adhered to the ethical requirements of the Institutional Review Board (IRB) at National Taiwan Normal University.

## 4. Results

Based on the interview data, the responses of participants in the academic and artistic domains were analyzed separately. The data were categorized and organized according to factors across multiple levels, including the individual, family system, learning system, and sociocultural system. Finally, the interactions among these factors were further examined.

### 4.1. Academic Domain

Based on interviews with nine participants in the academic domain of the preschool gifted program—language (S11, S12, S25, S27), mathematics (S03, S13, S14, S28), and science (S30)—the factors influencing career decision-making were identified and analyzed.

#### 4.1.1. Personal Factors

The findings of this study indicate that most students were able to identify their interests and career directions, develop an understanding of themselves and their careers, and engage in them with a high level of commitment.

(1) Thorough understanding and commitment
  Before making career decisions and committing to a particular path, S12 and S30 actively sought to gain a comprehensive understanding of the current state and future development of their chosen fields. S12 engaged directly with work in the field to assess personal suitability, while S30 emphasized that career decisions should not be based on blindly following others’ advice and, therefore, explored the field through multiple approaches.
(2) Agency in career decision-making
  Gozali and Paik (2023) found that gifted individuals tend to maintain outstanding performance in both education and employment, with primary motivations in adulthood including the desire to create impact (57.89%) and the drive for success (23.68%). S03, S13, S27, and S28 all indicated that they pursue what they enjoy without being constrained by family or societal expectations. S25 and S28 noted that their advanced abilities expanded their opportunities and freedom of choice. Although S30 followed a developmental path consistent with societal expectations of gifted individuals, they still aspired to create alternative possibilities.
(3) Pragmatic approaches to career decision-making
  Dai and Li (2023), in their study of scientific talent, found that individuals tend to explore their niches and optimize their developmental pathways. Similarly, S14 and S25 were able to identify their niches when making career decisions, taking into account available resources, future prospects, and desired lifestyles. Although S27 demonstrated strong athletic performance, they did not consider becoming a professional athlete as a career option; instead, they regarded sports as a personal interest and pursued alternative career paths.
(4) The influence of unexpected events
  Sometimes, career development does not proceed according to plan. Although S03 had a smooth academic journey and was originally offered a direct-entry PhD opportunity at Stanford University, the passing of his father and the impact of the COVID-19 pandemic led him to choose employment instead, thereby altering his original career plan.

#### 4.1.2. Family System

Parental support plays a significant role in adolescents’ career decision-making. H.-P. Yu (2002) found that parents of gifted students provide guidance and advice regarding career choices, offer behavioral support, and supply necessary assistance and information. Freeman (2013) noted that many children tend to adopt their parents’ perspectives; even when a career path may not be well suited to them, they may still accept it as reality. However, in the present study, most parents of the participants assumed supportive and facilitative roles, demonstrating respect for their children’s choices without imposing pressure.

(1) Family support and assistance
  S03 and S11 reported having positive relationships with their families, with parents who were open-minded, respectful of their decisions, and supportive in various ways. S13’s parents assisted in gathering career-related information, while S28’s parents encouraged and financially supported studying abroad, shared their experiences, and helped broaden his worldview.
(2) Parental expertise and guidance
  Parental professional backgrounds often exert a subtle yet significant influence on children. S12’s father, a physician, provided opportunities for him to transform his interests into professional development. S14 noted, “*A large part of my computer skills came from my father. My mother works in the financial sector, which made me more sensitive to numbers and more interested in the financial industry, eventually influencing my choice of graduate studies.*” He clearly recognized that his career decisions were shaped by his parents’ professional backgrounds.

#### 4.1.3. Learning System

Certain talents, such as those in science and mathematics, tend to receive greater encouragement in school settings; however, in the absence of appropriate guidance, young individuals may risk wasting time and effort (Freeman, 2013). In addition, within the school context, peers—alongside teachers—play an important role by providing emotional support, serving as role models, and offering feedback, all of which can influence career exploration and decision-making (Huang et al., 2015).

(1) Opportunities for further study provided by schools
  While pursuing graduate studies, S14 became acquainted with an overseas professor, which led to opportunities for further study abroad.
(2) School expectations and guidance
  S11 attended a gifted program in high school, which allowed her to engage in specialized learning at an early stage. She noted, “*The learning experience in the high school social sciences gifted program helped me realize that I am not someone who enjoys purely static, academic inquiry… and I was able to recognize early on that I did not have strong academic aspirations in certain humanities subjects.*” This experience enabled her to better understand her aptitudes and interests.
  In contrast, although S27’s high school imposed academic expectations, it lacked sufficient guidance for further education. He stated, “*The expectation for me was to perform well in what I was good at, such as studying and grades, but no one really guided me on which direction I could choose.*” As a result, he selected a major that did not align with his interests and abilities.
(3) Overseas learning experiences
  Opportunities for overseas learning allowed S11 to gain a deeper understanding of international work environments, which influenced her attitudes and values toward work. Through this experience, she became more independent and developed greater self-efficacy. Similarly, S30’s university provided opportunities for international exchange and internships, broadening her range of career options.
(4) Peer influence
  During junior and senior high school, S28 lacked peers with similar career aspirations, noting that “*it felt like my peers did not grow up in the same kind of environment.*” As a result, he experienced a lack of belonging and did not have companions with whom he could strive toward shared goals.

#### 4.1.4. Sociocultural System

(1) Alignment of career direction with mainstream values
  For gifted students in academic domains, career development often aligns with dominant societal values, which are internalized and influence their choices. S27 perceived his decision as consistent with mainstream expectations, stating, “*It seems that what society considers an appropriate path, along with what my family and I want, overlaps to some extent… you can clearly see that everyone expects you to follow this path.*” As a result, he did not experience significant resistance or conflict.
(2) Influence of societal expectations
  Societal expectations and values may also lead to inequalities in students’ choices and opportunities. Although S12 believed she had a stronger passion for the arts, she ultimately chose a safer career path.
(3) Identification with societal values
  While societal values may shape career decisions, S25 suggested that such considerations can also be meaningful, helping individuals identify better developmental opportunities and pathways.

The findings from the academic domain suggest that career decision-making among gifted individuals is influenced by multiple interacting factors, including personal, family, learning, and sociocultural systems.

At the personal level, most participants showed a clear understanding of their interests and abilities and were able to make decisions with a strong sense of commitment. Many of them actively explored possible career paths and considered both personal preferences and practical concerns. In some cases, unexpected life events also influenced the direction of their career development.

At the family level, parents generally played a supportive role by providing advice, resources, and encouragement. Rather than directing their children’s choices, most parents allowed them to make their own decisions, while still offering guidance when needed. In addition, parents’ professional backgrounds sometimes shaped participants’ familiarity with certain fields.

Within the learning system, schools provided important opportunities such as academic training, overseas experiences, and interactions with peers. These experiences helped participants better understand their interests and expand their career options. However, some participants also reported a lack of clear guidance in making educational or career choices. Peer relationships were another important factor, influencing both motivation and sense of belonging.

At the sociocultural level, many participants’ career choices were consistent with mainstream social expectations. These expectations were often internalized and contributed to relatively smooth decision-making processes. At the same time, they may have also limited the range of options considered by some participants.

Overall, the findings indicate that career decision-making in academically gifted individuals develops through the combined influence of multiple systems rather than being shaped by a single factor.

### 4.2. Artistic Domain

Based on interviews with nine participants in the artistic domain of the preschool gifted program—fine arts (S09, S34, S35), music (S17, S32, S33), bodily–kinesthetic (S36), and artistic (S39, S41)—the factors influencing career decision-making were identified and analyzed.

#### 4.2.1. Personal Factors

Sansone and Thoman (2005) suggested that students with strong artistic talents often experience dilemmas in career decision-making due to considerations of family expectations and future livelihood; such moments also serve as opportunities for reflection on personal interests and self-awareness. The findings of this study indicate that gifted students in the arts indeed take practical considerations into account when making career decisions. In addition to their abilities and interests, they also evaluate factors such as career prospects and stability. Furthermore, they consider personal values and preferred work environments as important references in their decision-making.

(1) Understanding of one’s abilities and interests
  - Choices under multiple abilities: S09 and S34 both possessed diverse abilities and interests, yet they were able to understand their own strengths and characteristics and accordingly determine their career directions. S34 stated, “*The way of thinking in law aligns with how I think—this is where I truly feel a match*” and thus chose to pursue a career in law. S39, on the other hand, recognized that teaching was not suitable for her: “*I don’t think my speaking skills are that strong, and I get nervous. But I wanted a relatively stable job, so I eventually chose to become a civil servant.*”
  - Balancing interest and reality: Although S09 had a strong interest in music, practical considerations, such as stability and future prospects, influenced career decisions. Similarly, S39 transformed her talent into a personal interest and leisure activity: “*My interest is actually in dancing, but I realized that in choosing a job, dancing might not be very applicable… so I consider it more as an interest.*”
(2) Personally valued factors
  - Personal aspirations: Individuals differ in what they seek in their careers. S33 pursued a sense of achievement and recognition from others; S34 valued diverse exploration, stating a desire “*to pursue more possibilities in life.*” S35, having grown up in a high-pressure educational environment, noted: “*Since the schools I attended for elementary, middle, and high school were high-pressure environments, I experienced a lot of stress from an early age. Due to that, I wanted a job with less stress and a better work–life balance.*” Thus, she placed greater importance on work–life balance.
  - Environmental expectations: In addition to personal aspirations, participants also had expectations regarding their work environments. S32 emphasized interpersonal interactions in the workplace, while S35 prioritized alignment between personal and organizational values: “*When job hunting, I paid special attention to the values of the companies I applied to. During the interview process, I made sure the company’s values aligned with mine.*”
(3) Practical considerations
  - Balancing distance from and commitment to a career: S35, influenced by early exposure to Hollywood culture, became aware of the risks and limitations faced by women in the workplace and thus maintained a certain distance in career choices. S36, recognizing the demanding nature of performance careers, chose not to pursue performance as a primary career option.
  - Expectations for future quality of life: S33 prioritized future income and socioeconomic status and therefore chose to study medicine.
  - Sense of urgency: S41 felt dissatisfied with her university admission outcome and, driven by a sense of urgency, continuously sought further learning and self-improvement to compensate. She described herself as rebellious, noting that only practical and economic realities would influence her career choices.
(4) Limitations in career decision-making
  - Limited understanding of self and occupational fields: Due to limited exposure to career options, S41 experienced uncertainty in decision-making. She relied on trial and error and others’ experiences, using a process of elimination to identify her career path.
  - Lack of passion, motivation, and direction: S32, an individual with autism currently working in an organization that supports artistic creation among autistic youth, reported a lack of passion and motivation in his current work: “*I think this job is somewhat challenging, but the challenge is that I feel I lack passion for artistic creation…*” He also expressed uncertainty and even pessimism about his future: “*From my perspective, society seems to be in a rather stagnant state, and even if you continue to develop yourself, it may be difficult to achieve anything. So I would rather not set goals for myself.*”
(5) Opportunities provided by significant others
  S17, who is visually impaired, possesses exceptional musical talent but has faced limitations in career development due to visual impairment and health conditions. Fortunately, through the recommendation of a piano teacher, he was able to join a choir for individuals with visual impairments, which provided him with important opportunities for development.

#### 4.2.2. Family System

Research has shown that parents’ active involvement during children’s developmental years—by providing bounded freedom, encouraging independence, setting reasonable discipline and expectations, and conveying warmth—has a positive influence on children’s career development (Baumrind, 1971; Csikszentmihalyi et al., 1993; Gozali & Paik, 2023; Ryan & Deci, 2000). Recent studies further suggest that family support and parental career-related behaviors are positively associated with career adaptability, identity exploration, and confidence in navigating future career uncertainty (Guan et al., 2018; Wang & Dong, 2024). Moreover, parental sacrifices may foster gratitude and become an important motivational force for continued effort and persistence (Gozali & Paik, 2023).

The findings of the present study further indicate that beyond parental involvement and participants’ desire to meet family expectations, important roles were also played by family conditions, parental occupational backgrounds, and sociocultural values in shaping career decision-making processes. In particular, family expectations often influenced how participants evaluated socially valued, stable, or prestigious career pathways, especially among academically gifted participants. In contrast, artistically gifted participants more frequently experienced tensions between personal aspirations and family concerns regarding economic stability and future career uncertainty.

(1) Family support and advice
  Support for interests: S17’s family consistently supported her musical development: “*My mother has always encouraged me to prioritize music… since childhood, I felt suited for a music-related career, although I was not sure what exactly I could do.*” Her family even considered opening a café to provide her with a performance platform. Although S36 chose a major that did not align with her family’s expectations, her family still offered support, allowing her to feel secure: “*My parents may not have fully encouraged it, but at least they did not oppose it, so I did not feel much pressure from my family when making my choice.*”
  Guidance for development: In addition to emotional support, families also provided career-related advice. For instance, S17 created YouTube videos based on her family’s suggestions.
(2) Family involvement
  - Conflict: Differences in career perspectives between S35 and her family led to tension: “*This influence on my life definitely diminished as I moved out of home, and I would say I have a bit of a rebellious streak—or more accurately, a desire to live my life as I want rather than as others would like me to.*” The conflict subsided after she left home. Similarly, S09 noted that family involvement affected his sense of agency in career decisions: “*Their advice may have acted as a kind of resistance. If I had chosen to major in English from the beginning, my life trajectory might not have been so influenced by following others, and I might have had greater autonomy.*”
  - Fulfilling expectations: S33 chose to study medicine partly to meet her family’s expectations: “*Part of it is that I know I have enough support to do what I want, but at the same time, I cannot let go of their expectations.*” However, because her family’s expectations did not conflict with her own choices, this reduced potential tension.
(3) Family context
  - Family environment: Both of S39’s parents worked in government institutions, leading her to value stability in her career choices: “*I wouldn’t consider starting a business—that kind of risk is too high. I prefer a stable job with a pension, perhaps a nine-to-five position.*”
  - Single-parent family: S41 grew up in a single-parent household following her parents’ divorce, which fostered her independence and self-reliance. She stated, “*The most influential factor was that in this family, you have to be independent to survive, which is also why I am less influenced by external factors.*”
  - High-achieving family members: The accomplishments of S41’s family members also influenced her: “*In this environment, I realized that financial resources and knowledge are far more powerful than I had imagined.*” This motivated her to pursue graduate studies.
(4) Parents’ occupations and professional backgrounds
  Parents’ professions can shape children’s familiarity with and selection of career paths. S39 was influenced by her mother when choosing her university major and later by her father when selecting a type of occupation. S41, due to her family’s occupational background, had limited exposure to careers in other fields. In contrast, S34’s parents respected her interests and learning choices; although they were professionally accomplished, they did not expect her to follow in their footsteps.

#### 4.2.3. Learning System

Gifted students with artistic talents often possess diverse interests and abilities, requiring schools to provide rich learning opportunities and resources. However, when making career decisions, they may be influenced by high self-expectations, aspirations for the future, and sensitivity to others’ opinions. They may also experience confusion due to social environments and prevailing societal values (Chen & Zhang, 2015).

(1) Schools providing diverse opportunities
  An open and flexible school culture can support the development of students’ diverse interests. For example, S34 did not confine herself to a single field but explored multiple domains, including the arts, languages, neuroscience, and law, continuously expanding her possibilities. During her university studies, exposure to criminology and opportunities to interview incarcerated individuals led her to choose law as her career path.
(2) Resistance to academic pressure
  Due to the pressure of academic advancement, S09 lacked opportunities to continue developing his artistic interests during high school. As a result, the greater freedom experienced in university led to reduced academic engagement. He reflected, “*When I was taking courses in international affairs, if I had been more dedicated, I could have pursued a career as a diplomat.*” Consequently, he missed potential career opportunities.
(3) Peer influence
  - Mutual encouragement and learning: S41 benefited from peers in different fields, who provided opportunities for mutual learning and encouragement in career development.
  - Pressure from perceived differences: Although S36 had an interest in performance, she felt hesitant due to concerns about diverging from her peers: “*If I choose this path, it might be different from what others are doing, and that feeling can prevent me from making that choice.*” Such pressures, in turn, influenced her career decisions.

The findings from the artistic domain suggest that career decision-making among gifted individuals is shaped by a complex interplay of personal, family, learning, and sociocultural factors.

At the personal level, participants showed a strong awareness of their abilities and interests, but their decisions often involved balancing personal aspirations with practical concerns such as career stability and future prospects. Many participants did not directly translate their artistic talents into career choices, instead maintaining these talents as personal interests or secondary pursuits. In addition, some participants experienced uncertainty, lack of direction, or limitations in their understanding of available career options.

At the family level, parents generally provided emotional support, encouragement, and advice, while allowing a certain degree of autonomy. However, family expectations, values, and living conditions also influenced participants’ decisions. In some cases, differences in perspectives led to tension, while in others, alignment between family expectations and personal choices reduced potential conflict. Parents’ professional backgrounds and family environments further shaped participants’ exposure to and familiarity with different career paths.

Within the learning system, schools played an important role in providing diverse learning experiences and opportunities for exploration. These experiences helped participants broaden their perspectives and consider multiple career possibilities. However, academic pressure and insufficient career guidance sometimes limited their ability to further develop their artistic interests. Peer influence also played a role, offering both support and pressure, particularly when participants perceived themselves as different from others.

At the sociocultural level, participants were sensitive to societal expectations, economic realities, and perceived risks associated with artistic careers. These factors often led them to adopt more cautious or alternative career paths. While societal values sometimes constrained their choices, they also provided a framework for evaluating viable options.

Overall, the findings indicate that career decision-making in artistically gifted individuals tends to involve ongoing negotiation between personal interests and external constraints, resulting in more flexible and less linear developmental pathways.

### 4.3. The Interaction Between Individuals and Different Systems in Career Decision-Making

McMahon et al. were among the first to apply a systems theory framework to career development and counseling, categorizing the systems that influence individual career decision-making into personal, social, and environmental systems. Scholars have since emphasized that career decisions are the result of interactions within broader social contexts, with different systems dynamically influencing one another (Huang et al., 2015).

In the present study, interviews were conducted with gifted students in both academic and artistic domains, examining influencing factors across multiple systems, including the individual, family, learning, and sociocultural systems. The findings further reveal that the interactions among these systems contribute to different career decision-making outcomes among students.

#### 4.3.1. Personal and Family Systems

In terms of career decision-making, both academically and artistically gifted students emphasized the importance of family support and respect. Parents generally did not impose or require their children to follow specific career paths. However, students’ desire to meet parental expectations still influenced their choice of academic majors and career directions. For instance, S33 chose to pursue medicine because she felt unable to disregard her mother’s expectations, stating, “*Since I do not oppose my mother’s expectations, I can follow them—this way, both she and I can accept it.*” In contrast, S09 reported that following parental advice when selecting a major later became a constraint in his career development: “*When choosing my major, my parents told me not to choose English because everyone can speak English and I should choose something different… but in university, I ended up circling back to English, so their advice may have actually been a kind of resistance.*”

Parents’ professional backgrounds and resources often provide subtle yet advantageous developmental conditions. For example, S14’s parents’ professions exposed him to computers and finance from an early age, which subsequently influenced his academic choices. However, when parents’ professional backgrounds differ from their children’s intended career paths, this may limit students’ understanding of other industries, requiring them to engage in ongoing exploration and trial and error. As S41 noted, “*Because I didn’t know what I was good at or interested in, I just kept trying different things and used a process of elimination.*”

The influence of family on career development begins early in life. Parents’ values and sacrifices can become motivating forces that drive children to pursue achievement (Gozali & Paik, 2023), highlighting the critical role of family in career decision-making. Scholars have advocated for an “authoritative parenting style,” which emphasizes providing support without overprotection, allowing children to make their own decisions, and offering both practical and emotional support. This balanced approach—combining autonomy with guidance—can foster independence, initiative, leadership, and learning abilities (Gozali & Paik, 2023; Ryan & Deci, 2000). However, excessive parental intervention may lead to conflict due to differences in perspectives or a lack of understanding of specific fields. For example, S35 experienced conflict with her father due to differing views on career development.

Overall, the findings of this study support the adoption of an authoritative parenting approach in career decision-making, whereby parents provide support and resources while also allowing space for autonomy. Such an approach enables students to develop a stronger sense of self and make responsible career decisions.

#### 4.3.2. Personal and Learning Systems

Gifted students represent an important human resource for societal and national development. However, a common misconception is that students who demonstrate high academic achievement or exceptional talent are naturally capable of managing their future career development, possess clear career goals, and experience relatively little uncertainty regarding their developmental pathways. In reality, gifted students often encounter unique developmental challenges, including multipotentiality, identity exploration, high expectations, and uncertainty in career decision-making (Kerr & Sodano, 2003). Without appropriate career guidance and support, these challenges may complicate educational and career decision-making, leading some gifted students to experience difficulties in selecting pathways that align with their interests, abilities, and long-term goals (Kerr & Sodano, 2003; Napier et al., 2024).

Recent research has further emphasized that schools function not only as educational institutions but also as important developmental contexts influencing gifted students’ career values, aspirations, and future career choices. Within these contexts, educational experiences and relationships across school, family, and community settings may shape how gifted individuals construct future career possibilities and navigate developmental uncertainty (Napier et al., 2024).

In particular, supportive gifted education programs—including enrichment opportunities, acceleration, and differentiated curricula—may foster students’ long-term motivation, aspirations, self-concept, and career development trajectories (Reis & Renzulli, 2010).

Conversely, insufficient challenge, limited access to mentors, and inadequate career guidance may result in students wasting time and effort pursuing inappropriate developmental pathways or failing to fully realize their potential (Freeman, 2013; Olszewski-Kubilius et al., 2023). Therefore, career guidance for gifted students should move beyond traditional achievement-oriented approaches and instead support identity exploration, career adaptability, psychosocial development, and long-term talent development across diverse domains of giftedness.

Participants in the preschool gifted program generally attended high-quality schools, where their outstanding performance was recognized and valued, providing them with numerous opportunities. The rich learning experiences offered by schools fostered their enthusiasm for learning and work, while also enabling interaction with peers of similar abilities (Subotnik & Arnold, 1994).

For example, S11, who attended a high school gifted program, was able to engage in specialized learning at an earlier stage, which helped clarify her interests and career direction. S14 and S30 benefited from opportunities for overseas study and exchange internships provided by their schools, which broadened their perspectives on career choices. However, the absence of adequate career guidance in schools may also lead to inappropriate decisions or a lack of direction. S27 noted that due to insufficient career counseling in high school and an exclusive focus on academic performance, he made an unsuitable choice when selecting his university major.

For students in artistic domains, academic pressure often forces them to choose between academic performance and artistic development. Such pressure may lead to resistance, which, in turn, affects both their engagement in learning and their career decisions.

Peers also play a significant role in career decision-making. During adolescence, their influence can be comparable to that of parents, providing competition, motivation, and role models (Gozali & Paik, 2023). Positive peer relationships can foster mutual encouragement and learning; however, excessive concern about peer perceptions may also create pressure that influences career decisions. For instance, although S36 was interested in performance, concerns about choosing a path different from peers created psychological pressure that affected his decision-making. Conversely, a lack of peers with similar abilities may lead to a diminished sense of belonging. S28, who attended a relatively average high school, noted, “*If you want to study veterinary medicine, your classmates might think the required scores are too high… it feels like we didn’t grow up in the same kind of environment.*” Thus, one advantage of attending high-quality schools and universities is the opportunity to experience positive peer influence (Gozali & Paik, 2023).

Overall, the findings of this study suggest that rich and open learning systems can expand the scope of career decision-making. Appropriate career education is crucial, and high-quality schools can provide access to peers with similar abilities, thereby fostering positive peer influences.

#### 4.3.3. Personal and Sociocultural Systems

Career decision-making results from the interaction between individuals’ internal factors and external sociocultural contexts and environmental structures, involving multiple dimensions. Various systems—including environment, cultural expectations, social class, race, and gender—can all exert influence (Chen & Zhang, 2015). Since the 1990s, scholars have increasingly recognized the complex interplay of diverse forces in career decision-making, conceptualizing it as a macro-system that extends beyond family, school, and community to include family culture, social structures, and even national and global trends (Huang et al., 2015). If individuals’ potential can be identified, developed, and utilized—so that their work lies at the intersection of their talents, strengths, and passions—then their abilities can be effectively harnessed to address broader societal challenges.

In this study, students in the academic domain indicated that their career paths largely aligned with mainstream societal values and that these expectations, internalized over time, made career decision-making relatively smooth and free from significant conflict. S27 stated, “*What society considers appropriate, what my family wants, and what I want seem to overlap to some extent—it’s hard to say which is more influential.*” He attributed this to sociocultural influences, noting that it was evident that others expected him to follow a particular path. Similarly, S25 suggested that such societal values can, in some respects, be meaningful.

Most participants demonstrated a clear sense of their future direction and were not overly constrained by sociocultural influences. However, some chose practicality over personal interest, transforming their talents into hobbies or leisure pursuits. For instance, S09 described his decision between music and language: “*I am aware of my own limitations, so I placed music in the realm of interest. As for language, it is both my interest and strength, and also a more practical career option.*” Additionally, S35’s choices were influenced by gender and workplace culture: “*I still get starry-eyed from their projects, but that degree of removal from the actual industry affords me to dip my toes in this field that I hold a lot of love for while protecting myself from the lack of work–life balance and, to put it bluntly—nasty men in Hollywood!*”

Overall, the findings of this study highlight the subtle yet pervasive influence of sociocultural factors on career development. Societal expectations and realities shape career decision-making; however, when students possess a clear sense of direction and confidence in their abilities, they are able to exercise greater agency over their career paths.

This study integrates findings from both academic and artistic domains to examine gifted individuals’ career decision-making from a systemic and developmental perspective. To facilitate comparison, Table 2 summarizes the key factors influencing career decision-making across the two domains. The comparison between academic and artistic domains shows both shared patterns and important differences. Across both domains, participants demonstrated a strong sense of self-awareness and agency. They were generally able to recognize their abilities and interests and actively engage in the process of career exploration. In addition, career decisions in both groups were shaped by the combined influence of personal, family, learning, and sociocultural factors rather than any single determinant. These findings suggest that career development among gifted individuals is best understood as a process situated within multiple interacting systems. Despite these similarities, clear differences emerged in how career decisions were formed and carried out. In the academic domain, participants’ abilities were more likely to align with structured educational pathways and socially recognized career routes. As a result, their decision-making processes tended to be more stable, with fewer reported conflicts or uncertainties. Career development in this domain often followed a relatively direct progression, supported by institutional structures and societal expectations. In contrast, participants in the artistic domain faced a more complex decision-making context. Although they demonstrated strong artistic abilities, many did not pursue these talents as their primary career paths. Instead, they often balanced personal interests with practical considerations such as economic stability, job security, and long-term prospects. This resulted in more flexible and sometimes discontinuous trajectories, including shifts in direction or the maintenance of artistic engagement outside of formal careers. Another notable difference lies in the role of sociocultural influences. While participants in the academic domain tended to internalize mainstream expectations, which facilitated smoother decision-making, those in the artistic domain appeared more sensitive to perceived risks and uncertainties associated with their fields. This heightened awareness often led to more cautious or alternative choices. Taken together, these findings indicate that while gifted individuals share similar internal capacities—such as self-awareness and agency—the extent to which their talents are translated into career outcomes varies across domains. Career development in the academic domain appears to be more closely aligned with established structures, whereas in the artistic domain, it involves ongoing negotiation between personal aspirations and external constraints.

## 5. Conclusions and Implications

The findings suggest that career development trajectories differ substantially across domains of giftedness. Academically gifted individuals often experience greater alignment among personal abilities, family expectations, educational systems, and socially valued career pathways, resulting in relatively stable and linear developmental trajectories. In contrast, artistically gifted individuals more frequently encounter tensions between personal aspirations and external sociocultural expectations, leading to more nonlinear, exploratory, and uncertain developmental pathways.

The findings further indicate that gifted individuals’ career development is shaped through continuous interactions among personal aspirations, family systems, educational experiences, and sociocultural values across time. Rather than representing a fixed outcome of early giftedness, career development appears to be a dynamic, adaptive, and context-dependent process involving ongoing negotiation between individuals and their environments. Based on these findings, several implications for gifted education, career development, and family support are discussed below.

### 5.1. Implications for the Family System

Parents function not only as providers of emotional support and educational resources but also as important developmental systems that shape how gifted individuals perceive opportunity, risk, achievement, and socially valued career pathways. The findings of this study suggest that parental expectations and family values may significantly influence how gifted individuals construct their career identities and evaluate future possibilities.

For academically gifted participants, family expectations often aligned with socially recognized and achievement-oriented career pathways, contributing to relatively stable developmental trajectories. In contrast, artistically gifted participants more frequently experienced tensions among personal aspirations, economic concerns, and sociocultural expectations, suggesting that artistic career development may involve greater identity negotiation and perceived uncertainty.

Accordingly, family support for gifted individuals should extend beyond academic achievement and resource provision to include support for identity exploration, career adaptability, and autonomous meaning-making. Rather than directing children toward socially valued or low-risk pathways, parents may play a more constructive role by helping gifted individuals navigate uncertainty, explore multiple developmental possibilities, and negotiate the complex interaction between personal interests and sociocultural expectations.

### 5.2. Implications for the Learning System

Schools function not only as educational institutions but also as important developmental systems that shape gifted individuals’ perceptions of achievement, ability, and future career possibilities. The findings suggest that institutional structures, curriculum design, teacher expectations, and peer cultures may significantly influence how gifted students construct their career identities and evaluate socially valued career pathways. Educational environments that emphasize narrow definitions of achievement may inadvertently constrain students’ exploration of diverse career and developmental possibilities.

The findings further indicate that educational systems may provide uneven levels of structural support across different domains of giftedness. Academically gifted students often benefit from relatively clear educational pathways, institutional recognition, and socially validated career trajectories. In contrast, artistically gifted students frequently encounter greater uncertainty regarding future employment, economic stability, and social legitimacy. Consequently, schools should move beyond traditional achievement-oriented models and provide differentiated forms of career support that address domain-specific developmental challenges.

While academically gifted students may benefit from opportunities for advanced specialization, mentorship, and international learning experiences, artistically gifted students may require greater support for identity exploration, career adaptability, and navigating tensions between personal aspirations and sociocultural expectations. Schools should therefore create more flexible and diverse developmental environments that support gifted individuals in exploring multiple career possibilities rather than implicitly encouraging them to pursue only socially prestigious or economically secure pathways.

### 5.3. Implications for the Sociocultural System

Career decision-making is influenced by the interaction of multiple forces ranging from family and sociocultural contexts to broader global conditions. Gifted students may face heightened expectations and demands, which can increase the difficulty of making career decisions. The findings of this study indicate that most students demonstrate a strong sense of agency in their career choices; however, the extent to which their career paths align with mainstream societal expectations often determines whether they encounter greater conflict or barriers. This suggests that dominant societal values exert subtle yet pervasive influences on their decisions.

Social identity ascription (SIA) may shape individuals’ perceptions of their interests and abilities, highlighting the need for creative thinking in constructing and developing authentic career pathways. Therefore, it is important to cultivate and encourage students to draw upon hope, optimism, self-efficacy, and resilience when navigating rigid sociocultural values and constraints. By doing so, they can pursue career paths that align with their personal aspirations while ensuring that their careers reflect the intersection of their talents, strengths, and passions.

By adopting a systemic and developmental perspective, this study moves beyond viewing giftedness as an individual attribute, emphasizing instead how career pathways are shaped through continuous negotiation between the individual and the broader context.

## 6. Research Limitations

Regarding the research methodology, this study adopted a retrospective approach to examine the factors influencing career decision-making from early childhood to adulthood. However, because this study relied on participants’ recollections and narratives of past experiences, their accounts may inevitably have been influenced by memory limitations, selective recall, or personal interpretations of past events. As with all retrospective interviews, participants’ accounts may therefore reflect not only their past experiences but also their current identities, perspectives, and interpretations.

In terms of participants, this study focused on individuals who had taken part in a preschool gifted program. However, due to the passage of more than two decades, only 44 participants could be successfully contacted. Among them, three declined to participate in this study, resulting in 41 questionnaire respondents and 18 interview participants. Considering factors such as gender, domain of strength, and cohort, only 18 participants were invited for in-depth interviews. Because data from a substantial proportion of the original cohort were unavailable, it was not possible to determine whether systematic differences existed between participants and non-participants. In addition, participants’ current academic achievement, professional status, or personal circumstances may have influenced their willingness to participate in this study. For example, one individual declined participation due to severe depression, while two others declined through parental refusal. Some cases of non-participation may also have resulted from changes in contact information or other personal circumstances. Therefore, caution is required when interpreting the findings, as the results reflect only the experiences and perspectives of those who remained contactable and agreed to participate in this study more than twenty years later. Participants who chose to participate may also have held relatively more positive attitudes toward their gifted education experiences.

Finally, at the time of this study, some participants were still pursuing higher education, while others had only recently entered the workforce. Many described themselves as still being in an exploratory stage of development, suggesting that their achievements and career trajectories may continue to evolve over time. Accordingly, the findings primarily reflect career development during higher education and the early stages of employment. Future longitudinal follow-up studies are needed to gain a more comprehensive understanding of gifted individuals’ long-term developmental outcomes and career trajectories.

## Figures and Tables

**Table 1:** Current status of the interviewees.

No.	Cohort	Domain	Gender	Highest Educational Attainment	Employment Status	Interview Mode
S03	1	M	M	Massachusetts Institute of Technology (USA)	Tower Research Capital/quantitative research	Online
S09	1	F	M	National Chengchi University, Dept. of Diplomacy (minor: English)	Online English teacher	In person
S11	2	L	F	Sun Yat-Sen University (Guangzhou), international business	Brand marketing specialist, Chinese E-commerce company	Online
S12	2	L	M	University of Washington, biology	Veterinary assistant, Lake Union Veterinary Clinic	Online
S13	2	M	M	National Taiwan Normal University, math education	Teacher, Taipei First Girls High School (math)	In person
S14	2	M	M	National Yang Ming Chiao Tung University, finance	Preparing for PhD abroad	In person
S17	2	U	F	National Taiwan University of Arts, music	Choir member (visually impaired choir)	Online
S25	3	L	M	Taipei Medical University, dentistry	Dentist, Shuang Ho Hospital	Online
S27	3	L	M	NYCU School of Dentistry	Student	In person
S28	3	M	M	University of British Columbia, master’s in data science	Military service	Online
S30	3	S	F	National Taiwan University, medicine	Pediatric hematologist, NTU Hospital	In person
S32	3	U	M	National Taiwan University of Arts, dance	DoBao Studio/artistic creation	In person
S33	3	U	F	Kaohsiung Medical University, medicine	Student	Online
S34	3	F	F	Stanford Law School	Student	In person
S35	3	F	F	Wellesley College, double major in English and media arts and sciences	Marketing designer, Wistia	Online
S36	3	B	F	National Chengchi University, Dept. of Radio and Television	Social media editor	In person
S39	4	A	F	National Taiwan Normal University, Dept. of Technology—Design and Science Division	Preparing for graduate school exams	Online
S41	4	A	F	Fu Jen Catholic University, textiles and clothing	Preparing for graduate school exams	Online

Note: Letters representing strength domains: L = language, M = mathematics, S = science, U = music, F = fine arts, B = bodily–kinesthetic, and A = artistic. Reprinted from C.-H. Yu and Kuo (2026).

**Table 2:** Factors influencing career decision-making across different systems.

	Academic Domain	Artistic Domain
Personal Factors	Thorough understanding and commitment Agency in career decision-making Pragmatic approaches to career decision-making The influence of unexpected events	Understanding of one’s abilities and interests Personally valued factors Practical considerations Limitations in career decision-making Opportunities provided by significant others
Family System	Family support and assistance Parental expertise and guidance	Family support and advice Family involvement Family context Parents’ occupations and professional backgrounds
Learning System	Opportunities for further study provided by schools School expectations and guidance Overseas learning experiences Peer influence	Schools providing diverse opportunities Resistance to academic pressure Peer influence
Sociocultural System	Alignment of career direction with mainstream values Influence of societal expectations Identification with societal values	

## Data Availability

The original contributions presented in this study are included in the article. Further inquiries can be directed to the corresponding author.
